# Cytocompatibility of Ti–xZr alloys as dental implant materials

**DOI:** 10.1007/s10856-021-06522-w

**Published:** 2021-04-23

**Authors:** Pinghua Ou, Cong Hao, Jue Liu, Rengui He, Baoqi Wang, Jianming Ruan

**Affiliations:** 1 State Key Laboratory of Powder Metallurgy, Central South University, Changsha, 410083 PR China; 2 Department of Stomatology, Third Xiangya Hospital, Central South University, Changsha, 410013 PR China; 3 Department of Orthopedics, Xiangya Hospital Central South University, Changsha, 410008 Hunan PR China; 4 Hunan Province Key Laboratory of Engineering Rheology, Central South University of Forestry and Technology, Changsha, 410004 PR China

## Abstract

Ti–xZr (*x* = 5, 15, 25, 35, 45% wt%) alloys with low elastic modulus and high mechanical strength were fabricated as a novel implant material. The biocompatibility of the Ti–xZr alloys was evaluated by osteoblast-like cell line (MG63) in terms of cytotoxicity, proliferation, adhesion, and osteogenic induction using CCK-8 and live/dead cell assays, electron microscopy, and real-time PCR. The Ti–xZr alloys were non-toxic and showed superior biomechanics compared to commercially pure titanium (cpTi). Ti–45Zr had the optimum strength/elastic modulus ratio and osteogenic activity, thus is a promising to used as dental implants.

## Introduction

Tooth loss is a common problem that can weaken chewing function, and even lead to more serious complications such as alveolar bone atrophy, masticatory muscle atrophy, facial collapse, etc. Dental implants are routinely used in oral rehabilitation to replace lost teeth (unitary or total), and have improved patient quality of life [1]. Pure titanium (Ti) is a standard dental implant material owing to its excellent mechanical properties and good biocompatibility [2, 3]. However, the tensile strength of commercially pure titanium (cpTi) is low, which can result in implant fractures, especially in individuals that exhibit bruxism [4]. In addition, Wachi et al. reported that the Ti ions released from these implants can trigger peri-implant mucositis, which eventually progress to peri-implantitis accompanied by alveolar bone resorption [5]. Therefore, different metal alloys are being considered due to their enhanced mechano-physical properties, which can potentially improve the clinical performance of the implants [6]. For instance, the Ti–6Al–4V alloy is increasingly replacing cpTi on account of its greater strength [7] and enhanced mechanical performance [8]. However, it is biomechanically incompatible to the human bone due to threefold higher Young’s elastic modulus (~110 GPa compared to human bones), which results in the “stress shielding effect” on the supporting tissues [9]. Furthermore, the vanadium (V) ions released into the blood and urine [10] can initiate an inflammatory cascade leading to osteolysis [11, 12], and aluminum (Al) ions are frequently present in the brain tissues of Alzheimer’s disease patients, which indicates a neurodegenerative effect [13]. Therefore, there is an urgent need to synthesize novel alloys with high mechanical strength and biocompatibility, and low elastic modulus.

Zirconium (Zr), a transient element belonging to Group 4 (according to new IUPAC name) in the periodic table, is chemically similar to Ti and does not display any local or systemic toxic effects [14]. The elastic modulus of Zr is 88 GPa, which is closer to that of the bone. Abdullah et al. compared Ti and Zr implants in a rabbit model, and observed similar bone contact with both, although Zr had superior biomechanics due to lower elasticity. In addition, the Zr ions released from the implant further increased the bone response [15]. Kobayashi et al. first proposed the biomedical application of Ti–Zr alloys in 1995 based on their superior mechanical and tensile strength [16]. Furthermore, binary Ti–Zr alloys with Ti as the primary component have shown encouraging results as dental implants [17, 18], in terms of both biomechanics [19, 20] and bioactivity [21]. The binary Ti–Zr alloy with 13~17% Zr is currently marketed as an implant material under the trade name Roxolid. In addition, equiatomic Ti–Zr alloys have also demonstrated excellent mechanical properties and good corrosion resistance [22, 23]. Kobayashi et al. found that Ti–Zr binary alloys with 30–60% Zr have higher Vickers hardness and superior tensile strength [24], which was confirmed by another study that observed higher micro-hardness and bending strength of Ti–Zr alloy with 40% Zr (Ti–40Zr) [25, 26]. Teisuke et al. found that Ti–30Zr or alloys with >50% Zr were ideal for clinical applications in terms of corrosion resistance [27]. Taken together, Ti–Zr alloys are a mechanically improved alternative to cpTi with similar biocompatibility, and can reduce the risk of implant fracture.

However, the optimal proportion of Zr and Ti for dental applications has not been established. We recently prepared Ti–xZr alloys with varying amounts of Zr (*x* = 5, 15, 25, 35, 45% wt%) by combining powder metallurgy with heat treatment. The Ti–xZr alloys exhibited maximum bending strength of 867.1 MPa and maximum compressive strength of 1599.8 MPa, which are superior to that of cpTi. Furthermore, all Ti–Zr alloys showed a lower elastic modulus (53.5–59.3 GPa) compared to cpTi (103 GPa) [28]. However, the biocompatibility of these binary alloys also need to be determined for any future clinical applications. To this end, we analyzed the cytocompatibility and osteogenic activity of Ti–xZr alloys in vitro to ascertain the optimal Zr concentration for dental implants.

## Materials and methods

### Preparation of Ti–Zr alloys

The Ti–Zr alloys comprising of 5–45% Zr by weight were prepared by powder metallurgy as described previously [28]. Pure Ti and Zr were supplied by Zhongzhou Alloy Material Co. Ltd. The chemical composition is presented in Table 1.

**Table 1:** The impurities of in Ti and Zr powder

Powder	Purity (%)	C (wt%)	O (wt%)	N (wt%)	H (wt%)
Ti	99.5	0.065	0.245	0.0645	0.13
Zr	99.8	0.016	0.0751	0.0571	0.094

### Preparation of alloy specimens

Ti and Zr discs measuring 10 mm in diameter and 1 mm in thickness were polished using silicon carbide papers up to 1200 grit. After sequential cleansing with acetone, ethanol and deionized water under sonication for 15 min each, the specimens were sterilized in an autoclave.

### Ion release test

The alloy discs (three samples per group) were immersed statically in 6 ml modified SBF solution (pH 7.4) at 37 °C for 30 days. The specimens were removed, and the concentration of metal ions in the SBF solution was measured by Inductively Coupled Plasma-Atomic Emission Spectrometer (PROFILE SPEC, Leeman). The experiment was repeated three times.

### Cell culture

The MG63 human osteoblast cell line was provided by The Center for Advanced Research of Central South University, China. The cells were cultured in DMEM supplemented with 10% FBS and 1% penicillin-streptomycin at 37 °C, 5% CO_2_ and 95% humidity. The medium was replaced every 48 h, and the cells were passaged at 80% confluency following trypsin (0.25%) digestion, and re-seeded at the appropriate density for in vitro assays.

### Preparation of alloy extraction medium

The alloy specimens were immersed in complete DMEM and incubated at 37 °C, 5% CO_2_ and 95% humidity for 72 h. The ratio of the disc surface area to the extraction medium volume ratio was 3:1 in accordance with EN ISO standard 10993:12 [29]. The resulting 100% extraction medium was used for testing cell viability.

### Live/dead cell assay

MG63 cells were seeded in a 24-well plate at the density of 2 × 10^4^ cells per well and incubated for 24 h to facilitate attachment. After replacing the media with 1 ml 100% extraction medium, the cells were cultured for another 24 h, washed twice with PBS, and stained with 50 μl Hoechst 33342 (0.25%) and 50 μl PI (0.5%) at 4 °C for 20 min. The cells were then rinsed with PBS, and observed under a fluorescence microscope at the excitation wavelengths of 346 and 488 nm. The number of live and dead cells were counted in three random fields per well at ×10 magnification using the Image Proplus6 (ipp6) software.

### Cell Counting Kit-8 assay

The MG63 cells were seeded in a 96-well plate at the density of 1 × 10^4^ cells per well, and allowed to adhere for 24 h. The medium was replaced with 1 ml 100% extraction medium, and the cells were cultured for 1, 3, and 5 days, with fresh medium replenished every 2 days. To determine the viability of the cells, 20 μl of CCK-8 test solution (Cell Counting Kit-8; Dojindo, Japan) was added to each well, and the cells were incubated for another 1.5 h. The absorbance at 450 nm was measured using an ELISA microplate reader (Molecular Devices, USA). Sterile culture media was used as the negative control, and cells incubated in 100% cpTi extraction medium as the positive control. Each sample was measured four times.

### Acridine orange (AO) staining

MG63 cells were seeded on alloy surfaces at the density of 1 × 10^4^ cell per well in complete DMEM, and cultured for 6, 24, and 48 h. After washing twice with PBS, the cells were fixed with 95% alcohol for 10 min, air dried, and stained with 300 μl acridine orange (0.01%) per well for 5 min. The stained cells were rinsed with PBS for 1 min, cleared with 0.1 mM calcium chloride for 1 min, and rinsed again. The cells were observed under a fluorescence microscope at an excitation wavelength of 518 nm. The number of stained cells were counted in five random fields at ×10 magnification, using the Image Proplus6 (ipp6) software. The experiment was repeated thrice.

### Scanning electron microscopy (SEM)

MG63 cells were seeded on the alloy surfaces at the density of 1 × 10^4^ cells per well, and cultured in complete DMEM for 6 h, 48 h, and 9 days. The cells were fixed with 2.5% glutaraldehyde for 40 min, rinsed with PBS, and dehydrated through an ethanol gradient (25, 50, 75, 90, 95, 100%) for 15 min. After vacuum drying for 12 h, the specimens were sputter coated with 10 nm gold and observed under the Hitachi S-4800N field emission SEM.

### Real-time polymerase chain reaction (RT-PCR)

The MG63 cells were seeded on the different alloy surfaces at the density of 10,000 cells per well, and cultured for 3, 6, 9, and 12 days in complete DMEM. Total RNA was extracted from the cells using Omega R6934-01 Total RNA Kit II (BioTek, USA) and reversed transcribed to cDNA with the PrimeScript RT Master Mix (Takara) according to the manufacturers’ instructions. RT-PCR was performed using SYBR Premix Ex Taq II (Takara) on the CFX96 PCR System (Bio-Rad). Relative expression levels of Ki67, COLI-α1, ALP, OC, integrinβ1, and Cbfα-1 were calculated in terms of the comparative cycle threshold (Ct) as 2^(−ΔΔCt)^, with GAPDH as the internal control. The primer sequences are shown in Table 2.

**Table 2:** Primers used for RT-PCR analysis

Gene	Forward primer sequence (5′–3′)	Reverse primer sequence (5′–3′)
GAPDH	CGCTCTCTGCTCCTCCTGT	CCATGGTGTCTGAGCGATGT
Ki67	CCTACCTGGTCTTAGTTCCGT	GTTGGCGTTTCTCCTCTTTTC
COL1α1	CAACCTCAAGAAGTCCCTGC	AGGTGAATCGACTGTTGCCT
ALP	ACTCCAACGCTTACATCAT	CCACTCGTAGTTCTTCTCC
OC	TCAGAGATTTCTCCCGGATA	CGCCGCCGGCAGCTCCA
Integrinβ1	TCACCAAAGTAGAAAGCAG	CAAGGCAAGGCCAATAAGA
Cbfα1	GTCTTACCCCTCCTATCTG	GCCTGGCTCTTCTTACTGA

### Statistical analysis

The experimental data were expressed as the mean ± standard deviation of three replicates, and compared by two-way analysis of variance followed by Tukey’s multiple comparison test. *p* < 0.05 with a 95% confidence interval was considered statistical significance. GraphPad Prism 6 software (GraphPad Software Inc. La Jolla, CA, USA) was used for statistical analysis.

## Results

### Ti–xZr alloys are mechanically superior to cpTi

The mechanical properties of Ti–xZr alloys are summarized in Table 3, and indicate that the bending strength and compressive strength of the alloys increased with Zr content. Ti–45Zr exhibited the maximum bending and compressive strength values of 867.1 and 1599.8 MPa respectively, which are significantly higher compared to that of cpTi. The elastic modulus of the alloys ranged from 53.5 GPa for Ti–35Zr to 59.3 GPa for Ti–xZr, and were markedly lower than that of cpTi (103 GPa).

**Table 3:** Mechanical properties of cpTi and the Ti–xZr alloys (*x* = 5, 15, 25, 35, 45 wt%)

Alloys	Vickers hardness (HV)	Compressive strength (Mpa)	Elastic modulus (Gpa)
Ti	292 ± 6.23	786 ± 17.22	103 ± 3.28
Ti–5Zr	473 ± 10.17	1279.8 ± 20.28	59.3 ± 6.12
Ti–15Zr	481 ± 4.89	1367.9 ± 37.12	54.6 ± 5.57
Ti–25Zr	495 ± 8.26	1416.5 ± 22.36	53.5 ± 6.45
Ti–35Zr	513 ± 11.22	1435.8 ± 16.25	55.5 ± 8.37
Ti–45Zr	525 ± 9.08	1599.8 ± 37.21	53.9 ± 6.09

### Ti–xZr alloys are stable in vitro

The stability of the different alloys was evaluated by measuring the amount of ions released into the SBF. As shown in Table 4, less Ti or Zr ions were released from the Ti–xZr alloys compared to cpTi, which can be attributed to the formation of stable corrosion resistant films on the alloys.

**Table 4:** Ti and Zr ions in SBF

Alloys	Alloying elements	Ion concentration (μg/l)
Cp-Ti	Ti	0.003 ± 0.0007
Ti–5Zr	Ti	0.002 ± 0.0005
	Zr	0.001 ± 0.0002
Ti–15Zr	Ti	0.002 ± 0.0002
	Zr	0.001 ± 0.0003
Ti–25Zr	Ti	0.001 ± 0.0002
	Zr	0.001 ± 0.0002
Ti–35Zr	Ti	0.002 ± 0.0003
	Zr	0.001 ± 0.0001
Ti–45Zr	Ti	0.001 ± 0.0002
	Zr	0.001 ± 0.0003

### Ti–xZr alloys are biocompatible

**Fig. 1:**
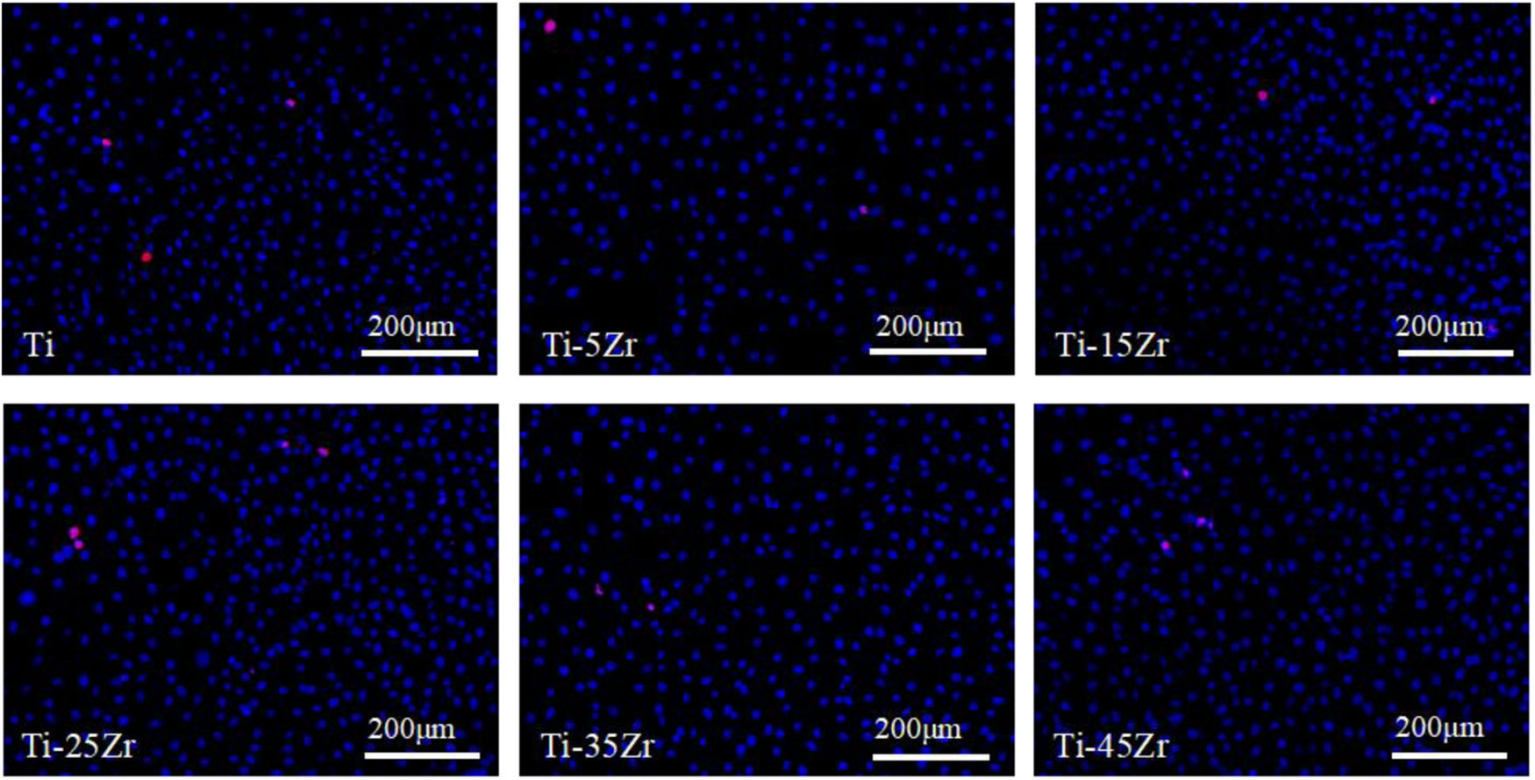
Representative fluorescence microscopy images showing Hoechst 3342 and PI-stained live (blue) and dead (red) cells after 24 h of incubation with different 100% extraction media

The biocompatibility of the Ti–xZr alloys was determined by analyzing the viability and proliferation of the MG63 cells grown in the alloy extraction media or on the different alloy surfaces. As shown in Fig. 1, very few dead cells were detected following 24 h incubation with the different 100% extraction media, and ~97% cells were viable. In addition, the viability was slightly higher in the Ti–xZr alloys compared to the cpTi group.

**Fig. 2:**
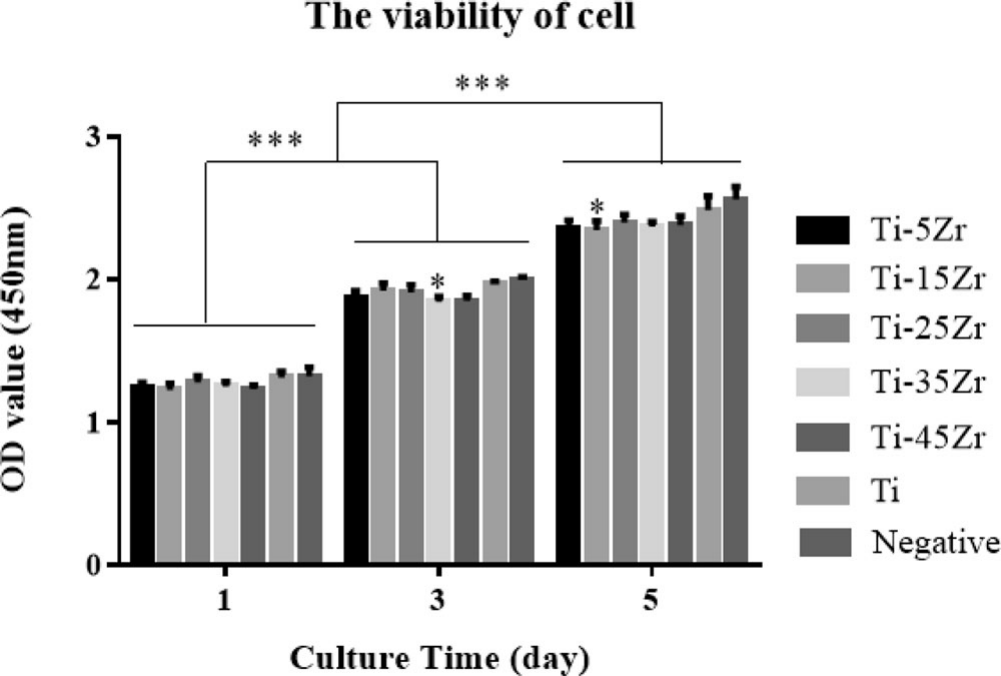
CCK-8 assays of the proliferation of cells cultured in 100% extraction medium of Ti–xZr alloys (5, 15, 25, 35, and 45% Zr) and cpTi on days 1, 3, and 5, with cpTi as control. Data are expressed as mean ± SD (*n* = 3). *p < 0.05, ***p < 0.001

The proliferation rates of the MG63 cells cultured in the different 100% extraction media were also measured over a period of 5 days. As shown in Fig. 2, the number of cells increased steadily in a time-dependent manner in all groups, and the proliferation rate was ∼80% on day 5 as compared to day 1 (*p* < 0.001). Furthermore, the viability and proliferation rates of cells cultured in the 100% extraction media of all but the Ti–15Zr and Ti–35Zr alloys were comparable to that of cells grown in cpTi extraction medium (*p* < 0.05).

**Fig. 3:**
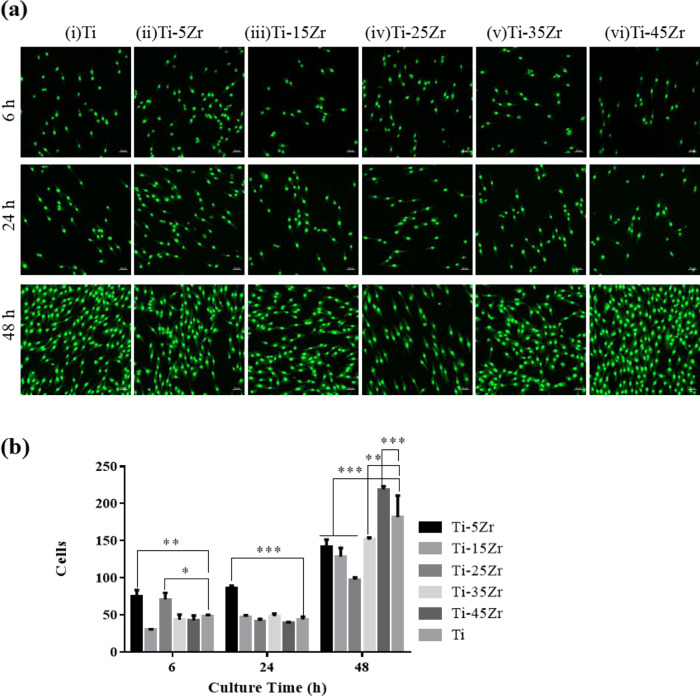
**a** Representative fluorescence microscopy images showing acridine orange-stained MG63 cells cultured for 6, 24, and 48 h on (i) Ti, (ii) Ti–5Zr, (iii) Ti–15Zr, (iv) Ti–25Zr, (v) Ti–35Zr, and (vi) Ti–45Zr. Scale is 50 μm. **b** The number of viable cells at the different time points in the indicated groups. Data are expressed as mean ± SD (*n* = 3). *p < 0.05; **p < 0.01; ***p < 0.001

Consistent with the above, the MG63 grown on the surface of the different alloys were also viable over a period of 48 h. As shown in Fig. 3a, the cells were able to attach to the metal surface within 6 h, and proliferated rapidly for 48 h. Furthermore, the total number of cells at 6 and 24 h were significantly higher on Ti–5Zr samples compared to the others at each time point (**p < 0.01). The cell count increased significantly in all groups by 48 h, and was highest on Ti–45Zr (***p < 0.001), and least on the Ti–25Zr alloys (***p < 0.001) (Fig. 3b). Taken together, the Ti–xZr alloys are non-toxic and can support the growth of osteoblasts, and Ti–45Zr shows greater biocompatibility compared to cpTi.

### The Ti–xZr alloys supported cellular adhesion and spread

**Fig. 4:**
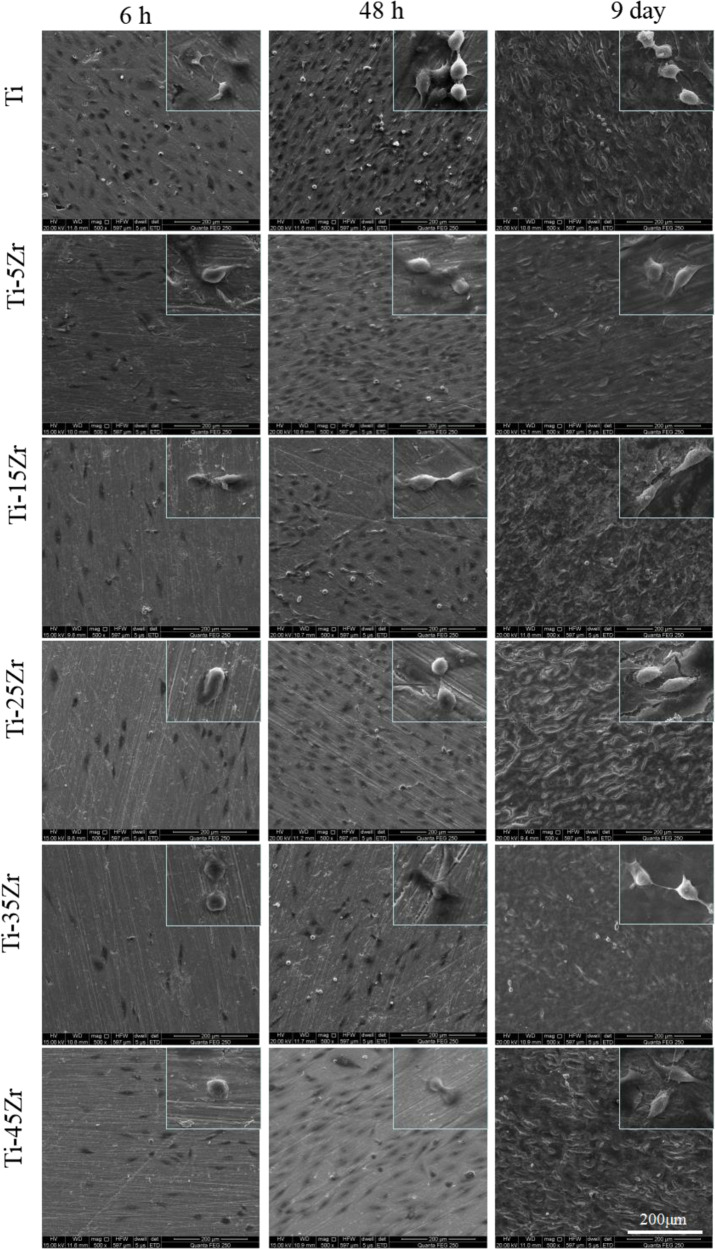
Representative SEM images showing the morphology of cells frown on different surfaces for 6 h, 48 h, and 9 days

The adhesion, differentiation, and migration of the MG63 cells on the cpTi and different alloy surfaces were analyzed by SEM. As shown in Fig. 4, the cells adhered to all surfaces within 6 h, and exhibited typical ellipse-like morphology while covering ~40–50% of the area. By 48 h, the cells had acquired a spindle-like morphology with uniform spread and prominent filopodia extending in all directions. In addition, ~70% of the surface area of all specimens except that of Ti–35Zr was occupied with the growing cells, which also showed considerable overlap indicating healthy growth. After 9 days, the surfaces of all specimens were completely covered by a highly dense cellular monolayer with multiple filopodia-like intercellular connections protruding from the edges of cells. The filopodia were particularly extensive along the leading edges of the cells. Thus, the Ti–xZr alloys allow osteoblast adhesion and growth comparable to that on cpTi.

### Ti–xZr alloys induced osteogenic differentiation

**Fig. 5:**
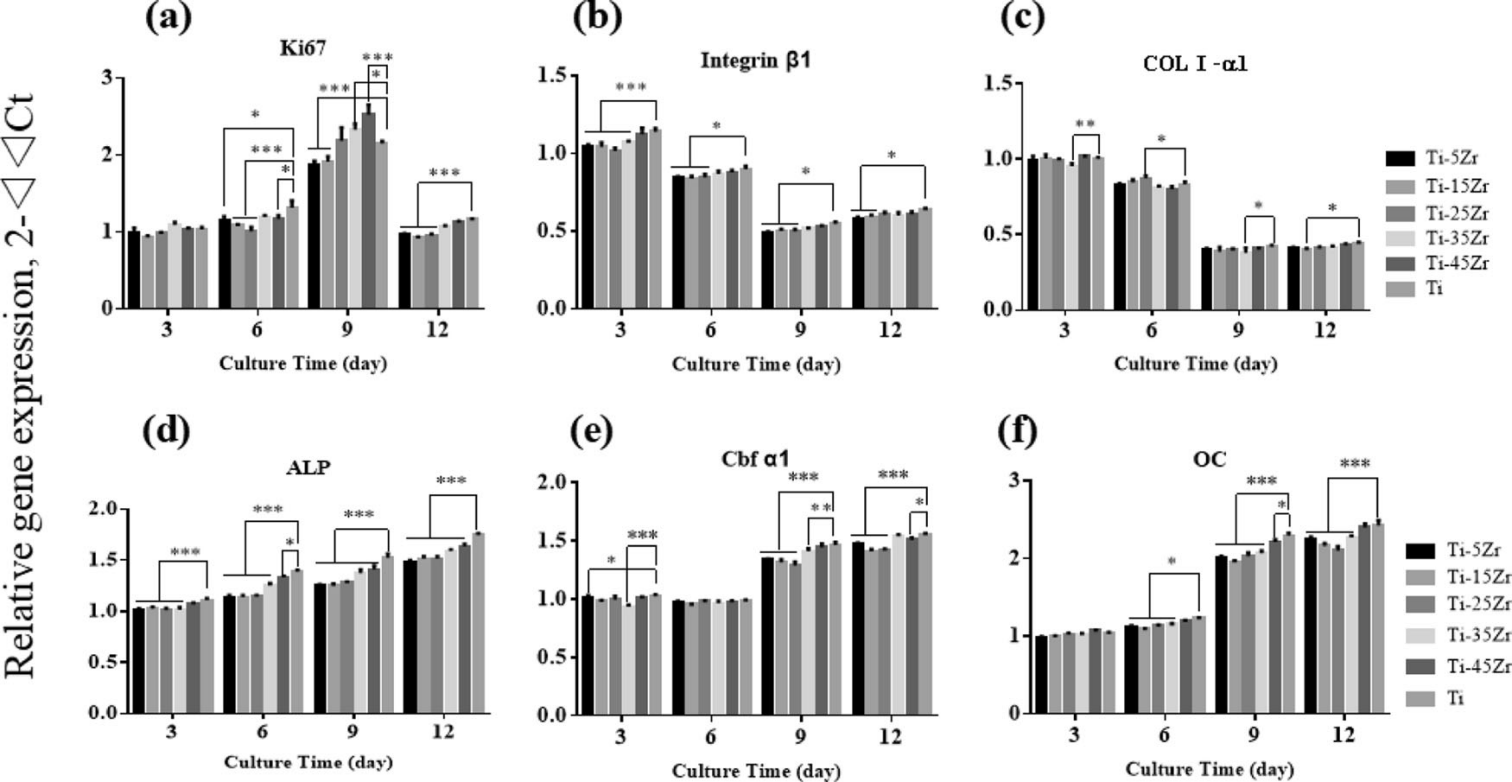
The induction of osteoblast differentiation-related genes in MG63 cells cultured on the different surfaces. Relative expression levels of (**a**) Ki67, (**b**) Integrin β1, (**c**) COLI-α1, (**d**) ALP, (**e**) Cbf α1, and (**f**) OC in the indicated groups. Data are expressed as mean ± SD (*n* = 3). *p < 0.05; **p < 0.01; ***p < 0.001

The osteogenic capacity of the different alloys was determined by analyzing the expression levels of osteogenesis-related genes in MG63 cells cultured for 3, 6, 9, and 12 days on cpTi and Ti–xZr alloys. As shown in Fig. 5c, COLI-α1 levels peaked on the 3rd day of culture and declined thereafter in all groups, and was similar between cpTi and the Ti–xZr alloys except Ti–35Zr, which showed lower expression compared to cpTi. In contrast, ALP (Fig. 5d), Cbf α-1 (Fig. 5e), and OC (Fig. 5f) were significantly downregulated in the cells grown on Ti–5Zr, Ti–15Zr, and Ti–25Zr on days 9 and 12 compared to that in the cpTi group (*p* < 0.001). Interestingly, Ki67 levels were lower in the Ti–5Zr and Ti–15Zr groups compared to that in cpTi on day 9 (*p* < 0.01), but significantly higher in Ti–35Zr and Ti–45Z groups (*p* < 0.001) (Fig. 5a). Furthermore, integrin β1 levels in Ti–5Zr, Ti–15Zr, and Ti–25Zr were significantly lower relative to the cpTi group on day 3 (*p* < 0.05) (Fig. 5b). To summarize, Ti–45Zr showed the maximum osteogenic activity compared to the other alloys, almost comparable to that of cpTi.

## Discussion

The primary reason for dental implant failure is “stress shielding” due to the dissimilar elastic modulus of the implant material and bone tissue. To maximize longevity of the implants and avoid the stress shielding effect, it is essential to fabricate materials with high tensile strength as well as a low elastic modulus comparable to that of the surrounding bone tissue. Studies have shown that Ti–Zr alloys can achieve good osseointegration and survival of the osteoblasts [30–33]. However, casting these alloys into dental molds is technically demanding and economically inefficient. Therefore, powder metallurgy is increasingly being used to produce dental alloys [34–36] of Ti and Zr in a cost-effective manner [37]. We used powder metallurgy to prepare Ti–Zr alloys across a range of Zr concentrations that showed good mechanical properties, excellent corrosion resistance and low elastic modulus [28]. In the present study, we evaluated the biocompatibility and osteogenic capacity of these alloys to determine their potential as dental implants.

Biocompatibility is an essential criterion for a medical implant, which should not cause any thrombogenic, toxic, allergic, or inflammatory responses in vivo. Ti dental implants usually fail due to the allergic reaction to Ti ions that leach from the alloys, eventually leading to inflammation in the surrounding tissues [38, 39]. Wachi et al. reported that Ti ions are the causative factor of peri-implant mucositis, which can progress to peri-implantitis accompanied by alveolar bone resorption [5]. Therefore, dental implant materials with high corrosion resistance are urgently needed. We tested the corrosion of the Ti–Zr alloys in SBF, and detected only miniscule amounts of Ti and Zr ions in the solution after 30 days of incubation. The higher stability of Ti–Zr alloys compared to cpTi can be attributed to the formation of corrosion resistant oxide films on the alloy surface. Consistent with our results, Akimoto et al. found that 90% less Ti ions were released from Ti–30Zr compared to pure Ti after 1 week of immersion in physiological fluid. This indicates that the ZrO_2_ film is more stable and resilient to dissolution compared to the oxide of Ti, and addition of Zr can significantly reduce Ti ion dissolution [27]. Therefore, binary Ti–Zr alloys have gained considerable attention in biomedical applications on account of their higher mechanical strength [40, 41], corrosion resistance [42], and biocompatibility compared to cpTi [31, 43].

The cytocompatibility of the Ti–Zr alloys was evaluated in the MG63 osteoblast cell line. This line was originally isolated from human osteosarcoma, and retains the osteogenic characteristics along with the advantage of unlimited growth [44]. In recent years, they have completely replaced osteoblasts for analyzing the cytotoxicity of biomaterials in vitro [45]. However, in the study of cell morphology and osteogenic differentiation, MSCs cells with differentiation potential have more advantages [46]. The alloys were highly conducive to the survival and proliferation of MG63 cells, which is consistent with the general biosafety of Zr [47]. Cell adhesion to the implant surface is crucial for the subsequent growth, differentiation, and migration [48]. We found that the MG63 cells adhered to, spread and rapidly proliferated on the alloy surfaces. Consistent with our results, Saldana et al. also showed [49] that osteoblasts can attach to coarse-grain or fine-grain Zr surfaces, and subsequently spread, proliferate, differentiate, and mature to a similar extent as on the Ti6Al4V alloy. Furthermore, the ZnO_2_ film can also promote cellular adhesion, proliferation, and differentiation [50], thus underscoring the suitability of Ti–Zr alloys as surfaces for osteoblast growth [51]. The differentiation of osteoblasts into mature osteocytes involves proliferation, extracellular matrix maturation, and matrix mineralization. Integrin β1 and COL1 α-1 levels were similar across all groups during osteoblastic adhesion and early proliferation. In contrast, the osteogenic markers (Ki67, ALP, Cbf α1, and OC) were significantly upregulated in the late proliferation and maturation stages of osteoblast differentiation on cpTi and Ti–45Zr, whereas the alloys with higher Zr content showed stronger osteogenic activity, which could be attributed to the superior osteo-inductivity of Zr. A recent study showed that ZrO_2_ and Zr ions promoted the proliferation and differentiation of human osteoblasts in vitro [52, 53].

To summarize, Ti–xZr alloys have superior biomechanics compared to cpTi with high tensile strength and low elastic modulus, along with similar biocompatibility, which can reduce implant stress shielding and improve longevity. Ti–45Zr in particular showed the optimum strength/elastic modulus ratio and osteogenic activity, and is therefore a promising alternative to cpTi as a dental implant.

## Conclusion

The Ti–xZr (*x* = 5, 15, 25, 35, 45 wt%) alloys are stable and do not adversely affect osteoblast adhesion and proliferation in vitro. Ti–45Zr showed excellent cytocompatibility and osteogenic activity comparable to that of pure Ti. indicating its potential as a dental implant material.
